# Possible Superconductivity Transition in Nitrogen‐Doped Lutetium Hydride Observed at Megabar Pressure

**DOI:** 10.1002/advs.202409092

**Published:** 2024-11-27

**Authors:** Xingbin Zhao, Yu Huang, Shuailing Ma, Hao Song, Yanwei Cao, Hao Jiang, Yanping Huang, Tian Cui

**Affiliations:** ^1^ Institute of High Pressure Physics School of Physical Scientific and Technology Ningbo University Ningbo 315211 People's Republic of China; ^2^ Ningbo Institute of Materials Technology and Engineering Chinese Academy of Sciences Ningbo 315201 People's Republic of China; ^3^ State Key Laboratory of Superhard Materials College of Physics Jilin University Changchun 130012 People's Republic of China

**Keywords:** high‐pressure phase transformation, high pressure and high‐temperature method, materials science, nitrogen‐doped lutetium hydride, superconductivity

## Abstract

The pursuit of room‐temperature superconductivity at an accessible synthetic pressure has been a long‐held dream for both theoretical and experimental physicists. Recently, a controversial report by Dasenbrock‐Gammon et al. claims that the nitrogen‐doped lutetium trihydride exhibits room‐temperature superconductivity at near‐ambient pressure. However, many researchers have failed to independently reproduce these results, which has sparked intense skepticism on this report. In this work, a LuH_2±x_N_y_ sample is fabricated using high‐pressure and high‐temperature methods. The composition and structural characterization are the same as the aforementioned near‐ambient superconductor. In situ X‐ray diffraction investigations indicate that a high‐pressure phase transition toward *Fm* $\bar{3}$ *m*‐LuH_3±x_N_y_ occurred in the sample at 59 GPa. The temperature‐dependent resistance measurements reveal that two possible superconductivity transition are observed at 95 GPa, with *T*_c1_ ≈6.5 K for high‐*T*_c_ phase and *T*_c2_ ≈2.1 K for low‐*T*_c_ phase, arising from the disparate phases in the sample. Resistivity measurements in the *Fm* $\bar{3}$ *m*‐LuH_3±x_N_y_ phase under varying magnetic fields exhibited characteristics consistent with superconductivity, with an upper critical field *μ*_0_H_c2_(0) of 3.3 T measured at 163 GPa. This work is expected to shed some light on the controversy surrounding superconductivity in the nitrogen‐doped lutetium hydride system.

## Graphical abstract

Achieving room‐temperature superconductivity at accessible pressure remains a challenge. Research into nitrogen‐doped lutetium hydride (LuH_2±x_N_y_) has revealed that the possible superconductivity transition occurs at pressures above 95 GPa, shedding light on the ongoing controversy surrounding this material.

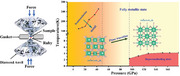

## 1 Introduction

The quest for room‐temperature superconductors at ambient conditions presents a formidable scientific challenge with vast potential for diverse applications. However, ever since its discovery in mercury at 4.2 K by Onnes,^[^ ^1^ ^]^ superconductivity has been achievable only at extremely low temperatures for a long time. Searching for higher *T*_c_ superconductors to break through low‐temperature limitations has been a primary enduring goal for both theoretical and experimental scientists. Since 2004, Ashcroft proposed the concept of hydrogen “chemical precompression” to pursue high *T*_c_ superconductors, hydrogen‐rich materials gradually emerging into public perspective.^[^ ^2^ ^]^ During the last decade years, the advent of hydrogen‐rich superconductors has revolutionized this paradigm, showcasing superconductivity properties at a relative high *T*_c_, which have emerged as a focal point of extensive research in condensed matter physics.^[^ ^3^, ^4^, ^5^ ^]^

At present, experimental efforts have made many high‐critical temperature hydrides, including binary hydrides as well as ternary hydrides. For instance, binary covalent hydride H_3_S, which has been theoretically predicted and experimental confirmation, exhibits a *T*_c_ exceeding 200 K under the pressure of 155 GPa.^[^ ^6^, ^7^, ^8^, ^9^ ^]^ Following this success, several binary superhydrides with high *T*_c_ above 100 K have been predicted and synthesized experimentally, such as LaH_10_ (250 K, 170 GPa),^[^ ^10^, ^11^, ^12^, ^13^ ^]^ CeH_9_ (115 K, 95 GPa),^[^ ^14^ ^]^ CaH_6_ (215 K, 172 GPa)^[^ ^15^ ^]^ and YH_9_ (243 K, 201 GPa)^[^ ^16^ ^]^ etc, which have reignited great enthusiasm on the explorations of rare‐earth hydrides to achieve room‐temperature superconductivity. Additionally, some researchers have opened up new hunting ground in ternary hydrides, which has more diverse chemical compositions, lower stabilization pressure, and richer structure prototypes than binary systems.^[^ ^17^, ^18^ ^]^ In recent experiments, nonstoichiometric alloyed ternary superhydrides (La, Al)H_10_,^[^ ^19^ ^]^ and (La, Ce)H_9‐10_^[^ ^20^, ^21^ ^]^ were experimental synthesized, showing a *T*_c_ of 233 and 176 K at moderate pressure, respectively. Moreover, Song et al. first synthesized the stoichiometric ternary hydrides LaBeH_8_ with a resolved crystal structure, and it exhibits a high *T*_c_ of 110 K at 80 GPa.^[^ ^22^ ^]^ Unfortunately, most of high *T*_c_ hydrogen‐rich compounds require extremely high pressures to achieve structural stabilization, which somewhat limits their practical application.

Quite recently, Dasenbrock‐Gammon et al. announced the discovery of room‐temperature superconductivity at 294 K under a remarkably low pressure of 10 kbar within the Lu‐N‐H system.^[^ ^23^ ^]^ However, this discovery has sparked intense debate within the scientific community. Notably, a series of experiments conducted by various researchers have failed to replicate the observed superconductivity in samples synthesized through alternative methods, raising questions about the reproducibility of the results.^[^ ^24^, ^25^, ^26^, ^27^, ^28^, ^29^, ^30^, ^31^, ^32^ ^]^ Later, Salke et al. independently characterized the electrical transport of the samples synthesized by the University of Rochester group, and they also reported the existence of a broader R(T) transitions in their samples.^[^ ^33^ ^]^ Then Denchfield announced that the superconductivity in Lu‐N‐H system could be influenced by N ordering in the structure.^[^ ^34^ ^]^ However, Wang et al.’s work examined the source of the resistance change near room temperature and determined that it was caused by a transition from a metal to poor conductor, rather than superconductivity.^[^ ^35^ ^]^ Hence, while current work tends to deny the superconductivity in Lu‐N‐H system at low pressure, there are exceptions.^[^ ^34^, ^36^ ^]^ Nevertheless, inspired by this work, many theoretical and experimental reports have also discovered high‐performance superconductors in the Lu‐H system. Du et al. had predicted stable superconducting compound in Lu‐H system under a pressure up to 400 GPa, and found the new superhydride *Pm* $\bar{3}$‐LuH_12_ with a high *T*_c_ of 291.3 K at 300 GPa.^[^ ^37^ ^]^ Li et al. also successfully synthesized the lutetium polyhydrides at high pressure and high‐temperature conditions, achieving a critical temperature of 71 K at 218 GPa.^[^ ^38^ ^]^

In view of many experiments have failed to observe superconductivity in nitrogen‐doped lutetium hydrides under low pressure, the question of whether it will exhibit superconductivity under relatively high pressure still remains. Previous theoretical studies have predicted that the superconducting phases in nitrogen‐doped lutetium hydrides may be thermodynamically unstable at ambient pressure, requiring high pressure for stabilization.^[^ ^28^, ^36^ ^]^ In this work, the LuH_2±x_N_y_ samples were synthesized using high pressure and high temperature (HPHT) method. The composition and structure of LuH_2±x_N_y_ are determined by X‐ray diffraction, EDS spectra, elemental mapping and Raman spectroscopy. Notable, the samples exhibit uniform dark‐bluish color at ambient pressure and undergoes four color changes within 61 GPa. Subsequently, we carried out the synchrotron X‐ray diffraction experiments on the samples, and found a structural transition toward *Fm* $\bar{3}$ *m*‐LuH_3±x_N_y_ occurred at 59 GPa. Finally, we carried out the electrical measurements from ambient pressure to 178 GPa, and observed two possible superconductivity transition occurred at 95 GPa, arising from the disparate phases (LuH_2±x_N_y_ and LuH_3±x_N_y_). These results display that achieving possible superconductivity in nitrogen‐doped lutetium hydride requires harsh conditions (extreme high pressure and low temperature).

## 2 Experimental Section

**Figure 1:**
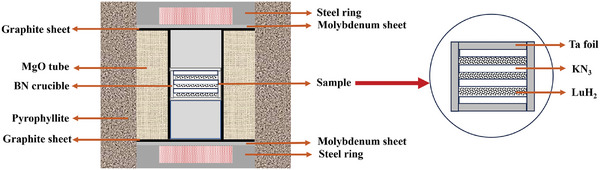
The schematic illustration of the assembly block in HPHT experiments.

The LuH_2±x_N_y_ samples were prepared by a reaction between LuH_2_ (purchased from Macklin, 99.9% in purity) and KN_3_ (Sigma–Aldrich, 99.9% in purity) using a HPHT method, which is one of several possible techniques to fabricate nitrogen‐doped lutetium hydride. For sample growth, we alternate placed the dense LuH_2_ and KN_3_ sample layers, and divided each layer with tantalum foil, preventing the contamination by potassium‐related compounds in the remaining products, as shown in **Figure** **1**. Then, the assembly blocks were processed in an SPD 6 × 600T cubic high‐pressure apparatus at the pressure range from 2 to 4 GPa, and the temperatures of 773–1273 K. Subsequently, the assembly block was smashed and selected the suitable samples in original LuH_2_ layer. The samples were characterized by X‐ray diffraction (XRD) using Mo Kα_1_ (*λ* = 0.7107 Å) radiation in Rigaku Nanopix WE X‐ray diffractometer (3 kW, 66 kV). The obtained XRD data were evaluated by the Rietveld method implemented using the GSAS‐EXPGUI software package.^[^ ^39^ ^]^ The sample exhibits a dark‐bluish when observed under transmitted white light, in accordance with findings reported by Dasenbrock‐Gammon et al.^[^ ^23^ ^]^ To clarify the nitrogen content in the as‐synthesized sample, the fracture surface was characterized by field‐emission scanning electron microscope (SEM, FEI Magellan 400L) coupled with energy‐dispersive X‐ray spectrometer (EDS). X‐ray photoelectron spectroscopy (XPS) analyses have been carried out using a surface science instruments spectrometer (ESCA LAB 250) with a focused Al Kα radiation (1486.6 eV) in an ultrahigh vacuum (UHV) chamber. Moreover, Raman scattering spectroscopy was collected with a 532 nm solid state laser on the Mono Vista CRS+ 500 Raman spectrometer system.

The high‐pressure synchrotron XRD experiments were conducted by a symmetric diamond anvil cell (DAC)^[^ ^40^ ^]^ at the BL15U1 beamline of the Shanghai Synchrotron Radiation Facility (*λ* = 0.6199 Å). The obtained images were converted to 1D diffraction using the DOIPTAS program.^[^ ^41^ ^]^ To understand the high‐pressure electrical transport behavior of the samples, four probe electrical resistance transport measurements were carried out using non‐magnetic Be‐Cu DACs in a multifunctional measurement system (CRYOMANETICS INC., 14TC‐MAG). Besides, the high‐pressure optical images were conducted by Leica microscope (M205C).

## 3 Results and Discussion

**Figure 2:**
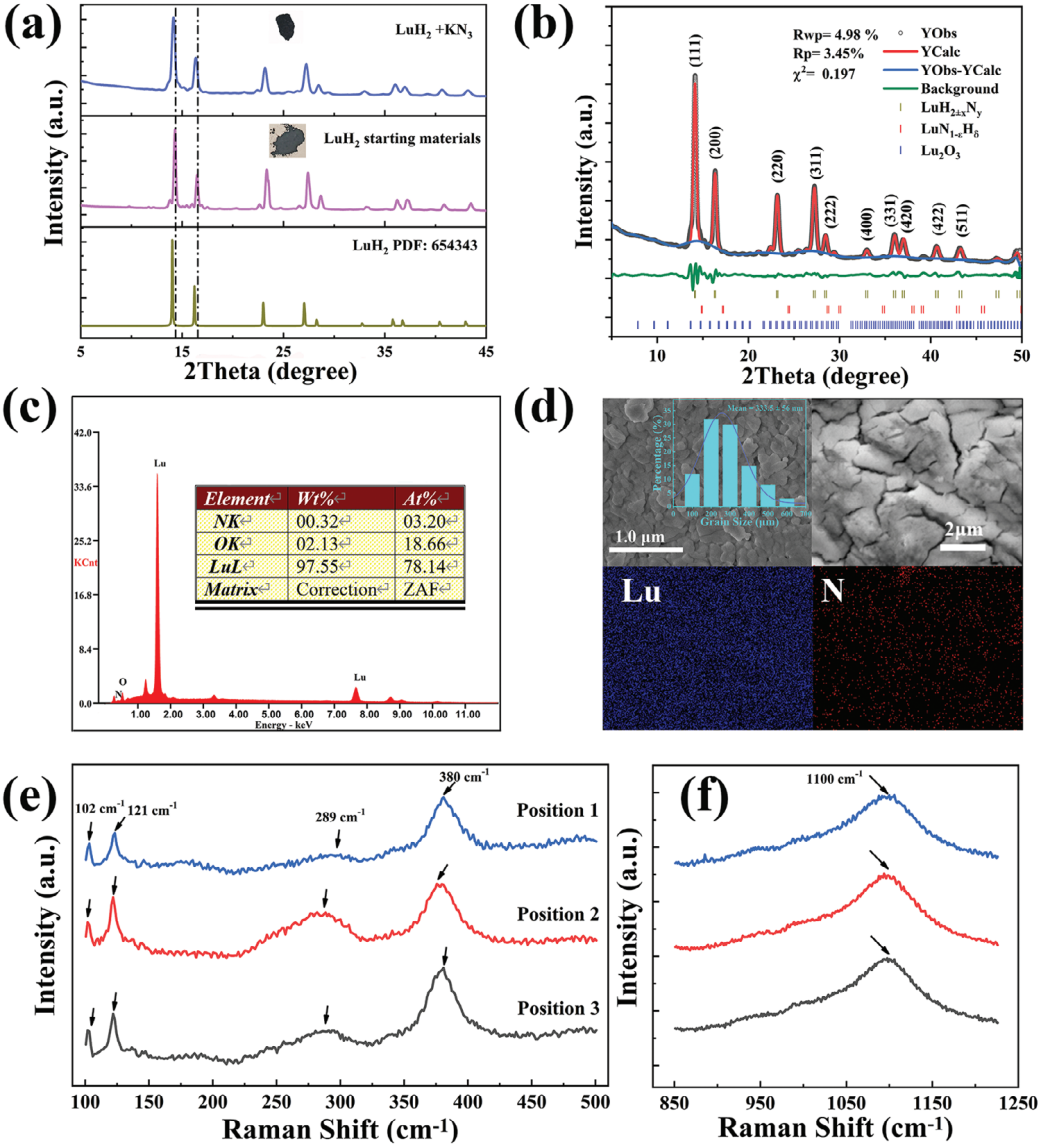
a) The XRD patterns of high‐pressure processed LuH_2_ and nitrogen‐doped lutetium hydride (LuH_2±x_N_y_) sample. b) Rietveld refinement on the XRD pattern of LuH_2±x_N_y_. The product is mainly composed of LuN_2±x_H_y_, and tiny amounts LuN_1‐ε_H_δ_ and Lu_2_O_3_. c) The EDS spectra of LuH_2±x_N_y_ on the polished surface. d) SEM images and typical elemental mapping of LuH_2±x_N_y_. e,f) are typical Raman spectra collected at ambient pressure for three different positions.

To further understand and confirm the crystal structure of LuH_2±x_N_y_ samples, we carried out the XRD measurements on the LuH_2_ raw materials and the samples, both of which were processed at 4 GPa and 1100 K, as shown in **Figure** **2**a. The XRD result revealed that the raw materials belonged to the reported *Fm* $\bar{3}$ *m* symmetry, with a tiny amount of Lu and Lu_2_O_3_ impurity. The slight right shift of peaks compared to the existing ICSD crystal information (PDF: 654343) comes from the lattice compression caused by high pressure. After the reaction between the LuH_2_ and KN_3_, the peak positions shifted ≈0.2° to a lower degree, which may be due to lattice expansion caused by nitrogen replacing hydrogen in the lattice. The inset of Figure 2a presents the raw LuH_2_ and our samples with dark‐bluish color. Figure 2b displays the Rietveld refinement results of the as‐synthesized sample, and the curve can be indexed by *Fm* $\bar{3}$ *m*‐LuH_2±x_N_y_ with an experimental lattice parameter *a* = 5.015 Å. The detailed crystal structure information of LuH_2±x_N_y_ is compiled in Table S1 (Supporting Information). Concerning impurities, there are a few small peaks cannot be fully matched, and they were identified as LuN_1‐ε_H_δ_ and Lu_2_O_3_, respectively. Comparing with the previously reported in Lu‐N‐H system, the structure of the as‐synthesized sample is highly similar to their results.^[^ ^23^, ^25^, ^30^ ^]^

Energy dispersive X‐ray spectroscopy (EDS) was used to detect the elemental composition of the LuH_2±x_N_y_ sample, as shown in Figure 2c. To minimize surface effects in EDS analysis, the sample underwent vacuum tube furnace pretreatment to remove dissolved oxygen and surface impurities. The representative EDS spectrum of the heat‐treated sample shows clear evidence for the incorporation of nitrogen in the sample. The nitrogen content derived from the results is ≈3.2%, which is significantly higher than previous reports.^[^ ^25^, ^30^ ^]^ Notably, the oxygen content detected by EDS remains relatively high, suggesting the need for further investigation into its origin. To delve deeper, XPS survey spectra were acquired before and after argon ion sputtering, as shown in Figure S1b (Supporting Information). These spectra reveal a significant decrease in oxygen content (≈90%) after sputtering to a depth of 120 nm, indicating that the high oxygen content is primarily confined to a thin surface layer and does not impact the bulk properties of the sample. Furthermore, XPS analysis after argon ion sputtering revealed distinct nitrogen and lutetium core level spectra, (Figure S1c,d, Supporting Information), confirming successful nitrogen doping within the LuH_2_ sample. This indicates that the nitrogen element detected by EDS originates from within the sample, not solely from surface contamination.

Further, we carried out the elemental mapping on the polished surface, as shown in Figure 2d, and found that the Lu and N elements were evenly distributed on the surface without any nitrogen aggregation. Figure 2d also gives the statistical grain size distribution of the sample derived from SEM analysis, and the average grain sizes is ≈335 nm. Besides, Raman‐scattering spectra was performed at ambient pressure with a 532 nm laser excitation, illustrated in Figure 2e,f. Distinct characteristic peaks were observed at ≈102, 121, 289, 380, and 1100 cm^−1^, respectively. These results are extremely similar to previously reported in Lu‐N‐H system. ^[^ ^23^, ^24^, ^30^ ^]^ The two low‐wavenumber peaks at 102 and 121 cm^−1^ are attributed to the influence of the Raman instrument itself, as confirmed by Ming et al.^[^ ^30^ ^]^ Besides, the Raman peaks at 289 and 1100 cm^−1^ can be attributed to the T_2_ _g_ mode in Lu‐H vibration, which are similar to the reported in LuH_2_ system.^[^ ^31^ ^]^ Interestingly, there is a new peak at 380 cm^−1^ observed only in this work, and it could be assigned to the N‐related vibrational modes.^[^ ^42^, ^43^ ^]^ The combined results of XRD, EDS‐mapping, and Raman spectra confirm the successful introduction of nitrogen into the as‐synthesized sample.

**Figure 3:**
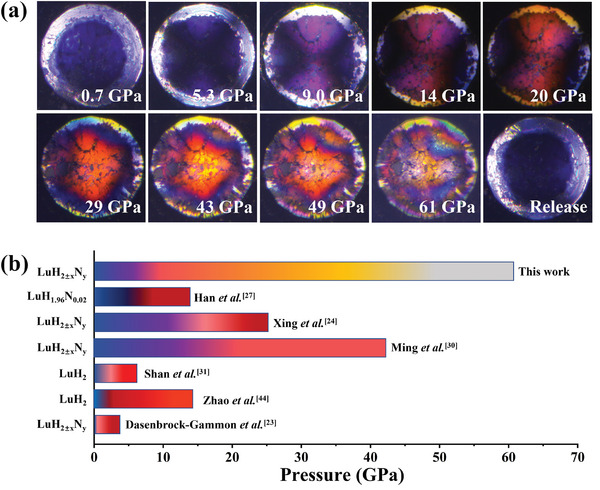
a) The color evolution of LuH_2±x_N_y_ sample with the pressure varying from 0 to 61 GPa. b) The sketch for pressure‐induced color changes in different Lu‐N‐H samples.

A key focus for LuH_2±x_N_y_ is the color evolution under pressure. **Figure** **3**a shows the color evolution of LuH_2±x_N_y_ up to 61 GPa, revealing four distinct color changes. The sample starts as dark blue, turns to purple at 5.3 GPa, similar to previous Lu‐N‐H samples,^[^ ^31^, ^32^, ^44^ ^]^ as shown in Figure 3b. As pressure increases, the color shifts from bright purple to orange ≈14 GPa, then yellow at 36 GPa, and finally silver gray at 49 GPa. Notably, the color changes are fully reversible during decompression.

**Figure 4:**
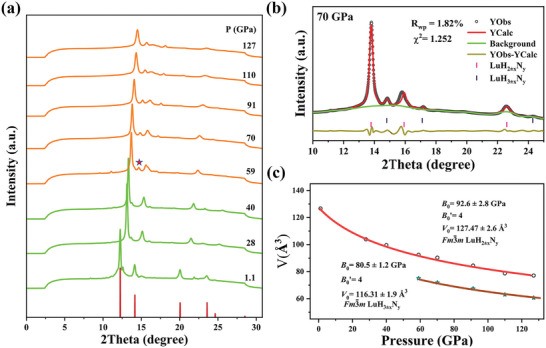
a) Synchrotron XRD patterns (*λ* = 0.6199 Å) for LuH_2±x_N_y_ sample at various pressures. b) The Rietveld refinement results of the sample at 70 GPa. Blue and pink vertical lines mark the position of the diffraction peaks for LuH_2±x_N_y_ and LuH_3±x_N_y_, respectively. c) The pressure‐dependent unit cell volume of LuH_2±x_N_y_ and LuH_3±x_N_y_. Red and yellow lines represent the fitting curves with the Birch–Murnaghan equation of state.

**Figure** **4**a presents the in situ XRD patterns collected up to 127 GPa at room temperature. As the pressure increases, all diffraction peaks continuously shifted toward higher angles. The original structure remains stable until 51 GPa, where a new peak appeared at ≈14.7°, signifying the onset of a pressure‐induced phase transition. Figure 4b presents the Rietveld refinement results of XRD pattern at 70 GPa. It is found that the new phase still belongs to *Fm* $\bar{3}$ *m* symmetry, and aligns well with the previously reported high_‐_pressure structure of LuH_3_.^[^ ^45^, ^46^ ^]^ We believe that high pressure induces a new phase *Fm* $\bar{3}$ *m*‐LuH_3±x_N_y_. To clarify structural evolution of as‐synthesized sample, we also plotted the unit cell volumes as a function of pressure based on XRD results, as shown in Figure 4c. The volumes of LuH_2±x_N_y_ and LuH_3±x_N_y_ both monotonically decrease with increasing pressure, by ≈39.1% and 19.2% at 127 GPa, respectively. Moreover, we also used the third‐order ($B_{0}^{\prime }$= 4.0) Birch–Murnaghan equation of state (EOS) to fit the pressure‐dependent unit cell volume,^[^ ^47^ ^]^ yielding bulk modulus *B*_0_ = 92.6 and 80.5 GPa, and zero‐pressure unit cell volume *V*_0_ = 127.47 and 116.31 Å^3^ for LuH_2±x_N_y_ and LuH_3±x_N_y_ respectively.

**Figure 5:**
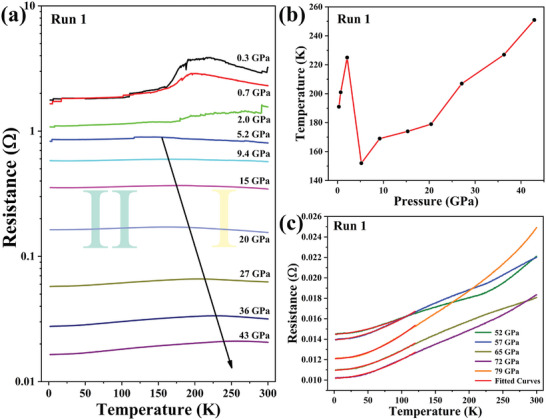
a) Typical temperature dependence of resistance for sample at the pressure ranges from 0.3 to 43 GPa. b) The evolution of the transition points for “phase I” and “phase II” with pressure. c) Temperature‐dependent electrical resistance at the ranges from 52 to 79 GPa. The red line represents the fitting result using the Bloch–Grüneisen formula.

Additionally, to investigate the electrical transition behavior of the samples, the temperature dependence of resistance curves were measured at different pressures from 0.3 to 79 GPa (Run 1). From **Figure** **5**a, the resistance at room temperature progressively decreases with increasing pressure, declining by two orders of magnitudes when the pressure reaches 43 GPa. Different from previous reports, we do not observe a superconducting‐related abrupt resistance drop or zero‐resistance state across the entire pressure range. These results do not support the room‐temperature superconductivity of Lu‐N‐H reported by Dasenbrock‐Gammon et al.^[^ ^23^ ^]^ Moreover, there is a semiconductor to metal transition as the temperature decreases under varying pressure, and we refer to as “phase I” and “phase II.” Below 2.0 GPa, a hump is observed, after where the resistivity rapidly decreased as the temperature decreased. With the pressure increases to above 5.2 GPa, the hump disappears and the semiconductor to metal transition becomes smooth. The transition temperature moves to higher values with pressure and the slope of R‐T curves approaches nearly zero (if not negative) at low temperatures. Figure 5b gives the evolution of transition temperature with pressure. It is clear that when the pressure exceeds 5.2 GPa, the transition temperature monotonically increases, reaching a maximum of 251 K at 43 GPa.

The reversible metal‐to‐semiconductor has been extensively observed in substoichiometric lanthanide trihydrides,^[^ ^48^, ^49^, ^50^, ^51^, ^52^, ^53^ ^]^ which is associated with the localization of the defect band at Fermi energy (E_F_), causing by the temperature‐dependent structural modification during warming.^[^ ^25^, ^50^ ^]^ Hence, owing to the similar crystal structure and resistance transitions, we speculate that the LuH_2±x_N_y_ sample in this work may share the same transition mechanism with other non‐stoichiometric hydrides.^[^ ^23^, ^54^ ^]^ In addition, when the pressure increases to above 52 GPa, the sample turns into a complete metallic behavior. Observe that this pressure precisely coincides with the point at which the hue transitions into a silvery gray. To further understand the electronic scattering mechanism of the sample at low region (6–120 K), we fitted the resistivity using the Bloch–Grüneisen formula,^[^ ^55^ ^]^ as shown in Figure 5c red lines. Here, the resistivity should exhibit a feature *R* (*T*) = *R*_0_  + *AT* ^α^ at low temperature range, where *A* and *α* are constant, and *R*_0_ is the residual resistance. The pressure dependence of *R*_0_ and the exponent *α* are shown in Figure S2 (Supporting Information). With the increase of pressure, the value of *α* various from 1.89 to 1.61, which is close to the value anticipated by the Fermi‐liquid behavior (2.0). Hence, we speculate that the metallic state behavior of LuH_2±x_N_y_ at high pressures originates from the phonon‐electron interaction.^[^ ^56^, ^57^ ^]^

**Figure 6:**
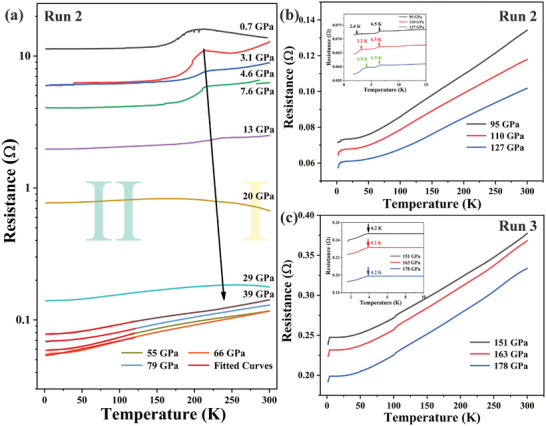
The typical temperature dependence of resistance in Run 2 at the pressure of a) ambient to 79 GPa, b) 95–127 GPa. The inset represents the enlargement of low‐temperature region. c) Temperature dependence of resistance and the enlargement of low‐temperature region in Run 3 at the pressure of 151–178 GPa.

Further, to better understand the electrical transition behavior in higher pressure, we conducted the temperature‐dependent of resistance measurement on another distinct sample at the pressure ranging from 0.7 to 127 GPa (Run 2). **Figure** **6**a shows that the measured resistance curves exhibit the same trend of variation below 80 GPa as Run 1, which supports our analysis of its electrical behavior as mentioned above. Surprisingly, as the continuous pressure increases at ≈95 GPa, we observe a sudden drop in resistivity at low‐temperature region, as shown in Figure 6b. We speculate that a superconducting transition may have occurred at this pressure. From the measurements of the electrical resistance curve, two possible superconducting transitions were observed with *T*_c1_ ≈ 6.5 K for high‐*T*_c_ and *T*_c2_ ≈ 2.4 K for low‐*T*_c_, which arises from the disparate phases LuH_2±x_N_y_ and LuH_3±x_N_y_, respectively. Moreover, we found that the *T*_c2_ decreased monotonically during compression, but *T*_c1_ has no obvious pressure dependence. Subsequently, we conducted Run 3 on third sample at 0.7–178 GPa, as shown in Figure 6c and Figure S3 (Supporting Information). When the pressure was increased to 151 GPa, the *T*_c1_ disappeared and *T*_c2_ reached to a maximum of 4.2 K, which is entirely contributed by LuH_3±x_N_y_. Regrettably, the zero‐resistance states did not occur until the pressure reaches 178 GPa, which may be caused by the low transition temperature and residual resistance of impurities.

**Figure 7:**
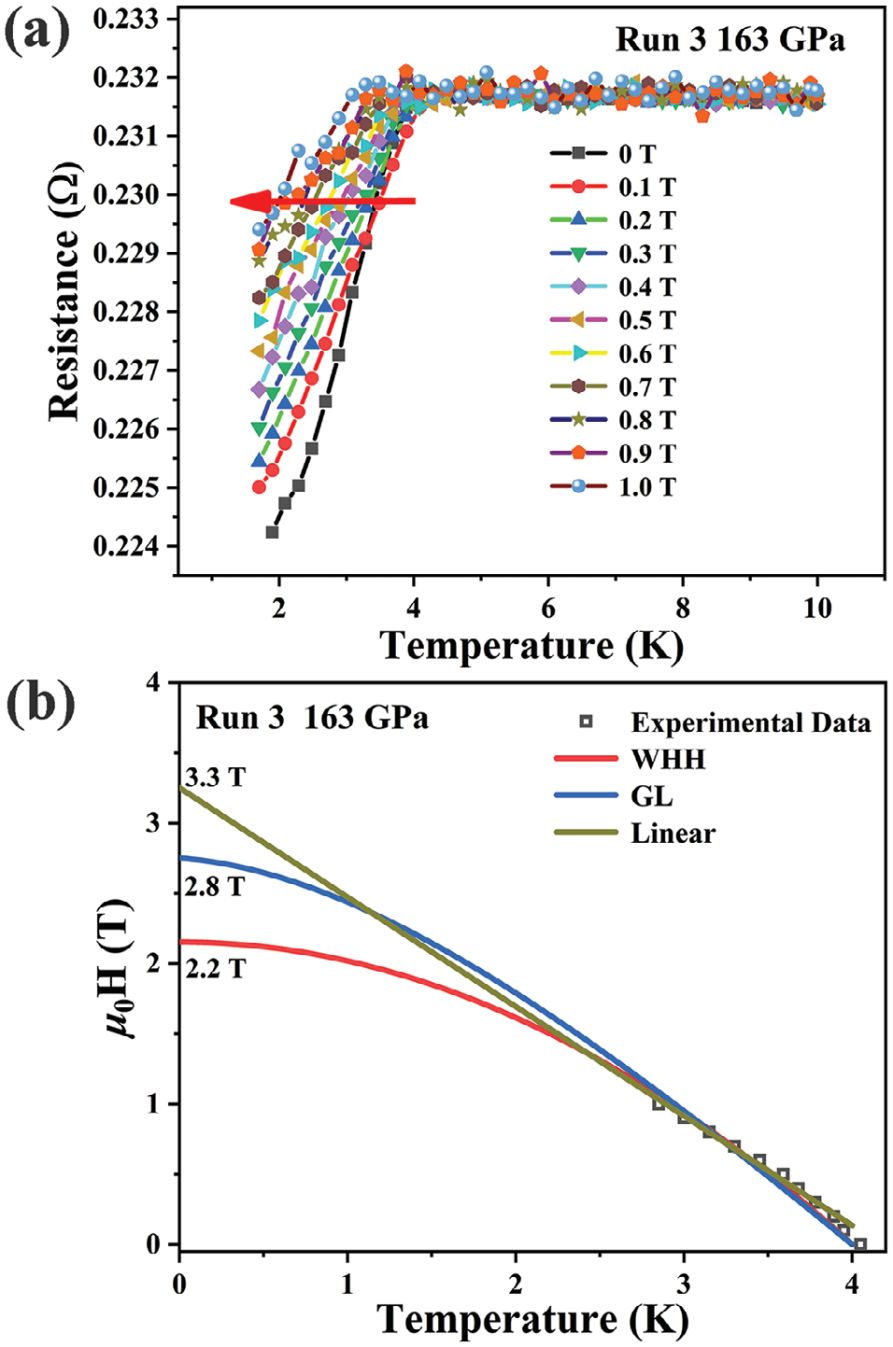
a) Temperature dependence of resistance in Run 3 measured at 163 GPa under different magnetic fields. b) The upper critical magnetic field is fitted by three models. Red, blue, and yellow line correspond to the WHH,^[^ ^58^ ^]^ GL,^[^ ^59^ ^]^ and linear^[^ ^60^, ^61^ ^]^ models fitting to the experimental data.

To gain a better understanding of the possible superconductivity in the samples, we performed low‐temperature resistivity measurements at 163 GPa while varying the external magnetic fields. As shown in **Figure** **7**a, with the increases of external magnetic fields from 0 to 1 T, the *T*_c_ is significantly suppressed, this observation is consistent with the expected behavior of a superconducting material. However, further experimental verification is recommended to conclusively determine the superconducting nature. To obtain the upper critical field *μ*_0_H_c2_(T), we fitted the experimental data using the linear model, Ginzburg–Landau (GL) model, and the simplified Werthamer–Helfand–Hohenberg (WHH) model.^[^ ^58^, ^59^, ^60^, ^61^ ^]^ As depicted in Figure 7b, the *μ*_0_H_c2_(T) values obtained by three model are 3.3, 2.8, and 2.2 T, respectively. Besides, according to the GL model, the corresponding coherence length *ξ* (0) is 10.8 nm fitted using the equation:

(1) $$H_{c2}=\Phi_{0}\;/\left(2\pi \;\xi^{2}\left(0\right)\right)$$

where *Φ*_0_ is the magnetic flux quantum constant (2.068 × 10^−15^ T·m^2^). To further understand the possible superconductivity behaviors of the sample, the Fermi velocity *υ*_F_ and carrier density *n* is estimated based on BCS theory:^[^ ^62^ ^]^

(2) $$\xi \;=\;0.18\hbar v_{F}/k_{B}T_{c}$$

(3) $$v_{F}=\sqrt{\frac{\hbar^{2}}{m_{e}^{2}}{\left(3\pi^{2}n\right)}^{\frac{2}{3}}}$$

The value of *υ*_F_ and *n* are 3.3 × 10^4^ ms^−1^ and 7.8 × 10^23^ m^−3^. Hence, the London penetration depth *λ*(0) can be estimated using the equation:

(4) $$\lambda \;\left(0\right)=\sqrt{\frac{\varepsilon_{0}m_{e}c^{2}}{ne^{2}}}\;$$

The *λ*(0) is approximately equal to 6013 nm, which yields the GL parameter as *κ* = *λ*(0)/*ξ*(0) ≈ 556.8, conforming to the typical features of a possible conventional type‐II superconductor.^[^ ^63^ ^]^

## 4 Conclusion

In summary, we have successfully fabricated the LuH_2±x_N_y_ sample using a HPHT method, and the structure, composition, and nitrogen content were confirmed by XRD, Raman, and EDS measurements. The pressure‐induced color evolution reveals that the sample has undergone four color changes at the pressure from ambient to 61 GPa. In situ, X‐ray investigations indicate the onset of a high‐pressure phase transition at ≈59 GPa, and pressure‐induced formation a higher hydrogen content *Fm* $\bar{3}$ *m*‐LuH_3±x_N_y_ structure. The resistance measurements under pressures up to 178 GPa reveal that the electrical behavior can be divided into three stages. From ambient to 43 GPa, there is a metal to semiconductor transition in LuH_2±x_N_y_ as the temperature increases, and the transition temperature monotonically increases with the pressure. When the pressure increases to 51 GPa, the sample turns into a fully metallic state, and the low‐temperature data indicates the appearance of Fermi‐liquid behavior. Finally, as the pressure increases to 95 GPa, we observe a sudden drop in resistivity, with two possible superconducting transitions *T*_c1_ and *T*_c2_. Besides, the low‐temperature resistivity measurements at 163 GPa under varying external magnetic fields consistent with the expected behavior of superconductivity in LuH_3±x_N_y_, with a maximum *μ*_0_H_c2_(T) of 3.3 T. LuH_3±x_N_y_ belongs to the typical type‐II superconductor with a GL parameter of 556.8. Our results delve deeper into the unsolved problems in the Lu‐H‐N system.

## Conflict of Interest

The authors declare no conflict of interest.

## Supporting information



Supporting Information

## Data Availability

The data that support the findings of this study are available from the corresponding author upon reasonable request.
