# Supplementary material for: Possible Superconductivity Transition in Nitrogen‐Doped Lutetium Hydride Observed at Megabar Pressure
Source: Adv Sci (Weinh). 2024 Nov 27;12(3):2409092. doi: 10.1002/advs.202409092 (PMC11744718; doi:10.1002/advs.202409092)
Supplement: Supplementary file 1 — Supporting Information [file ADVS-12-2409092-s001.docx]

**Supporting Information**

**Possible superconductivity transition of** **nitrogen-doped lutetium hydride observed at megabar pressure**

Xingbin Zhao^a,†^, Yu Huang^a,†^, Shuailing Ma^a^, Hao Song^a^, Yanwei Cao^c^, Hao Jiang^a^, Yanping Huang^a*^ and Tian Cui^a,b*^

^a^ Institute of High Pressure Physics, School of Physical Scientific and Technology, Ningbo University, Ningbo, 315211, People’s Republic of China

^b^ State Key Laboratory of Superhard Materials, College of Physics, Jilin University, Changchun, 130012, People’s Republic of China

^c^ Ningbo Institute of Materials Technology and Engineering, Chinese Academy of Sciences, Ningbo, 315201, People’s Republic of China

^†^ These authors contributed equally to this work

* Corresponding authors. E-mail addresses: [cuitian@nbu.edu.cn](mailto:cuitian@nbu.edu.cn); [huangyanping@nbu.edu.cn](mailto:yanpinghuang@nbu.edu.cn)

| **Table S1.** The details crystal structure information obtained from the Rietveld Refinement. | | | |
| --- | --- | --- | --- |
| Identification code | | LuH_2±x_N_y_ | |
| Crystal system | | Cubic | |
| Space group | | *Fm*$\bar{3}$*m* | |
| Unit cell dimensions (Å) | | *a* = 5.015(2) | |
|  | | *α* = *β* = *γ* = 90° | |
| Volume (Å^3^) | | 126.13 | |
| Atomic | Wyckoff | (x, y, z) | Occupancy |
| Lu | 4a | (0, 0, 0.) | 1 |
| H | 8c | (0.25, 0.25, 0.25) | 1-δ |
| N | 8c | (0.25, 0.25, 0.25) | δ |


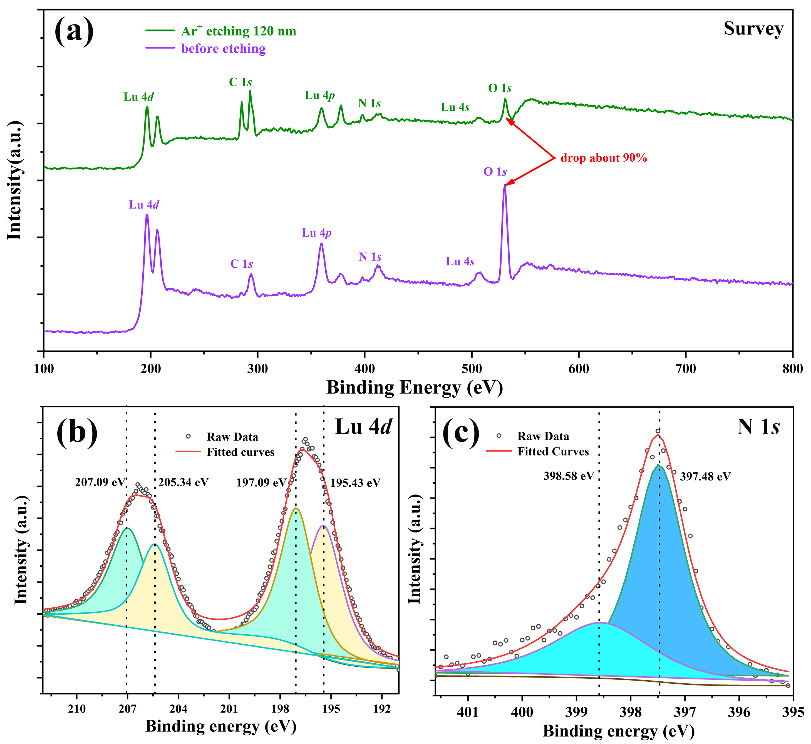


**Fig S1.** (a) The XPS survey spectra of LuH_2±x_N_y_ before and after Ar^+^ etching. (b) and (c) are the Lu 4*d* and N 1*s* core-level spectra of etched sample, respectively.





**Fig S2.** Pressure dependence of *R*_0_ and the exponent *α* derived from the fitting to $R\left( T \right)=R_{0}+AT^{\alpha}$ of LuH_2±x_N_y_ in low temperature region.


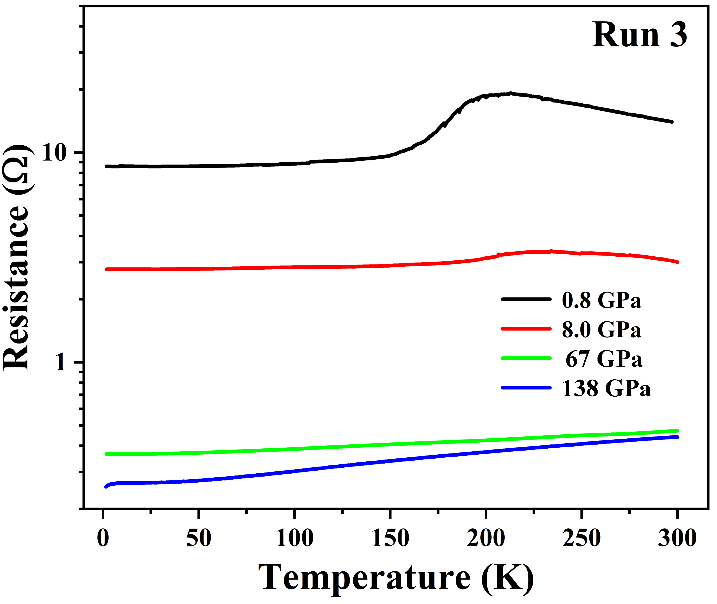


**Fig S3.** The typical temperature dependence of resistance in Run 3 at the pressure ranging from 0.8-138 GPa.

| **Table S2.** The details crystal structure information for high pressure phase LuH_3±x_N_y_ at 70 GPa. | | | |
| --- | --- | --- | --- |
| Identification code | | LuH_3±x_N_y_ | |
| Crystal system | | Cubic | |
| Space group | | *Fm*$\bar{3}$*m* | |
| Unit cell dimensions (Å) | | a = 4.158(3) | |
|  | | *α* = *β* = *γ* = 90° | |
| Volume (Å^3^) | | 71.89 | |
| Atomic | Wyckoff | (x, y, z) | Occupancy |
| Lu | 4a | (0, 0, 0.) | 1 |
| H | 4b | (0.5, 0.5, 0.5) | 1 |
| H | 8c | (0.25, 0.25, 0.25) | 1-δ |
| N | 8c | (0.25, 0.25, 0.25) | δ |
